# Central nervous system inflammatory demyelinating disorders of childhood

**DOI:** 10.4103/0972-2327.74204

**Published:** 2010

**Authors:** Mahesh Kamate, Vivek Chetal, Venkatesh Tonape, Niranjana Mahantshetti, Virupaxi Hattiholi

**Affiliations:** Department of Pediatrics, KLE University’s J N Medical College, Belgaum, Karnataka, India; 1 Department of Radiology, KLE University’s J N Medical College, Belgaum, Karnataka, India

**Keywords:** Acute disseminated encephalomyelitis, clinically isolated syndrome, CNS inflammatory demyelinating disorder, multiple sclerosis, neuromyelitis optica

## Abstract

**Background and Objectives:**

Childhood Central Nervous System (CNS) inflammatory demyelinating disorders (CIDD) are being diagnosed more commonly now. There is ambiguity in the use of different terms in relation to CIDD. Recently, consensus definitions have been proposed so that there is uniformity in studies across the world. The prevalence of these disorders and the spectrum varies from place to place. This study was undertaken to study the clinico-radiological profile and outcome of children with CIDD using the recent consensus definition.

**Study design:**

Prospective descriptive study.

**Materials and Methods:**

All patients admitted in pediatric ward and pediatric intensive care with neurological symptoms and signs suggestive of CNS inflammatory demyelinating disorders from July 2007–August 2008 were enrolled. The details of clinical presentation, neuroimaging findings, laboratory results, treatment, and outcome were noted and analyzed.

**Results:**

Fifteen patients (11 with acute disseminated encephalomyelitis and 4 with clinically isolated syndrome) were diagnosed with CIDD. Clinical presentation was quite varied. Eight patients recovered completely; 4 cases were left with sequelae and 3 patients expired. There were no cases of multiple sclerosis or neuromyelitis optica.

**Conclusions:**

CNS inflammatory demyelinating disorders are common illnesses in developing countries because of recurrent infections. Even the spectrum of CIDD is different. Neuroimaging in the form of magnetic resonance imaging is essential for diagnosis.

## Introduction

The CNS inflammatory demyelinating disorders (CIDD) of childhood include diseases like acute disseminated encephalomyelitis (ADEM), multiple sclerosis, transverse myelitis, optic neuritis, and others.[1] Different authors have used varying definitions for the disorders which come under the term CIDD. There is no clarity as to whether one condition evolves into another, how much to investigate these conditions and how to manage this. Though immunomodulatory treatment is used to manage these disorders, which agent to use and the duration of treatment is unclear. To reduce the ambiguity in definitions and to facilitate large multicentric studies, the International MS Study group in the year 2007, has proposed consensus definition for these inflammatory disorders.[1,2] The objective of the present study was to adopt this new consensus definitions and study the incidence, clinical profile, neuro-radiological features and outcome of children with CIDD. This would also encourage many more similar and larger studies from many other centers thereby providing with more concrete data on CIDD in children. The improved precision from the definitions and increased patient homogeneity would also facilitate international research.[1]

## Materials and Methods

All cases who presented with neurological symptoms and signs suggestive of CNS inflammatory demyelinating disorders (acute onset altered sensorium, focal deficits, seizures, abnormal movements, blindness, ataxia, tone abnormalities with or without fever) and admitted to the pediatric ward of a medical college hospital from July 2007–August 2008 were enrolled for the study. A detailed clinical history and examination was done by a pediatric neurologist and findings were entered in a pre-designed proforma. The patients were subjected to cerebrospinal fluid (CSF) analysis and neuroimaging. Magnetic resonance imaging (MRI) and Computer tomography (CT) scan films were read by an experienced radiologist, who was blinded to the patient’s identity and clinical presentation. The consensus definition was then applied to classify these disorders. They were managed with immunomodulatory therapy or careful observation depending on the lesion load on imaging and the clinical presentation. After discharge, all patients were regularly followed up at the pediatric neurology clinic and their recovery was recorded in the proforma. Ethical clearance was obtained from the institutional ethics board.

## Results

A total of 15 patients fulfilled the diagnosis of CIDD during the study period. The mean age at presentation was 5.6 years (Range: 10 months to 13 years). No sex predominance (M: F- 1:1) was found. Out of the 15 patients, 11 cases were diagnosed as acute disseminated encephalomyelitis (ADEM) and 4 cases as clinically isolated syndrome (CIS). There were no cases of multiple sclerosis or neuromyelitis optica in the present series. History of preceding viral exanthem was available in 6 patients, non specific febrile illness in 2, upper respiratory illness in 2, and gastroenteritis in 1 patient. Three of 15 patients had no preceding illness or vaccination.

**Figure 1:**
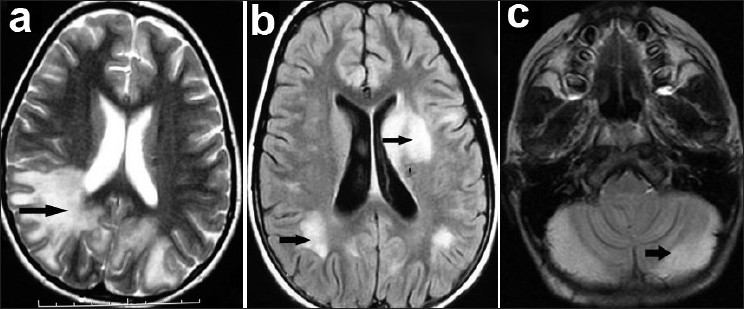
MRI of brain in CIDD. (a) T2-W axial image of ADEM patient showing hyperintense white matter signal changes in right parieto-occipital and left occipital lobes. (b) FLAIR axial cut of same patient showing hyperintense signals in left caudate nucleus, and subcortical white matter. (c) Axial FLAIR image of cerebellitis patient showing hyperintensity and swelling of both cerebellar hemispheres compressing the brainstem and occluding fourth ventricular outlet.

The common presenting features were described in Table 1. Presence of altered sensorium distinguished ADEM from patients with CIS. Other less common presenting features were headache and vomiting. Many of the cases of ADEM were referred to us with a diagnosis of encephalitis and few of them were on antiviral drug acyclovir as well. Cerebrospinal fluid analysis was done in 14 patients. It showed less than 10 cells in 10 patients, 10 to 50 cells in 2, and more than 50 cells in 2 patients (one patient had 120 cells). Proteins were elevated (>50mg %) in 6 patients (42.8%). Average CSF proteins were 80.5 mg% (Range:33± 190 mg %). MRI was done in 14 cases (one case of post infectious cerebellitis was diagnosed on clinical findings). White matter changes were noted mostly in frontoparietal areas in 8 (57%) and temporo-occipital area in 4 (28.5%) cases [Figure 1a]. Corpus callosal changes were seen in 1 patient (7%). Gray matter involvement (thalamus and basal ganglia) was noted in 2 cases (14%). Of 4 cases with CIS, 3 patients had evidence of cerebellitis [Figure 1b] and one patient had demyelinating plaque in the pons suggestive of brain-stem encephalitis. One child with cerebellitis had brainstem compression and communicating hydrocephalus. One of the patient with ADEM had a positive human immunodeficiency virus (HIV) ELISA test.

**Table 1:** Clinical presentation of patients with CNS inflammatory demyelinating disorders

Clinical features	Number (Percentage)
Altered sensorium	11 (73.3)
Seizures	8 (53.3)
Abnormal movements	3 (20)
Ataxia	4 (26.7)
Quadriparesis	1 (6.7)
Hemiparesis	2 (13.3)
Optic neuritis	2 (13.3)
Multiple cranial paralysis	3 (20)

Immunomodulatory therapy was initiated in 12 patients. Eight patients received pulse methylprednisolone (20–30 mg/kg for 5 days) and 2 patients received high dose dexamethasone (2–3 mg/kg/day for 5 days) because of non affordability. The patients who received dexamethasone tolerated it well and showed improvement like those on methylprednisolone. One patient (where we had diagnostic difficulty to rule-out viral encephalitis due to CSF pleocytosis) received intravenous immunoglobulin (IVIg) (2 gm/kg over 5 days), and one received both IVIg plus methylprednisolone as he did not respond to IVIg alone. Three patients improved without any kind of immunomodulatory therapy. Following pulse steroids, oral methylprednisolone (2 mg/kg/day) was prescribed in 4 patients for 2–4 weeks because of high lesion load on neuroimaging.

Following therapy, eight patients recovered completely over a period of 2–6 weeks; 4 cases were left with sequleae (2-hemiparesis, 1-quadriparesis, and 1 in minimally conscious state). Three patients expired (1 was HIV ELISA positive, 1 presented late in the course of illness, and the third child had fulminant cerebellitis with brainstem compression). A follow-up MRI was available in 3 patients who improved after an interval of 6–12 weeks. Lesions had disappeared in 2 patients and considerably decreased in size in 1 patient.

Patients who survived where followed up every 3 months in the first year and 6 monthly in the second year. The 8 patients who improved did not have any relapse. Out of 4 with sequelae, 1 patient who was in minimally conscious state improved to her pre-morbid state in the next 3 months and other 3 though had clinical improvement, continued to have sequelae (hemidystonia-1, generalized dystonia with oromotor dysfunction-1 and mild choreo-athetosis in one). MRI in 2 patients showed residual injury and had normalized in 2 patients.

## Discussion

CIDD includes both self-limiting and life-long conditions, which can be indistinguishable at the time of initial presentation. Majority of CIDD in childhood are monophasic ADEM, variants of ADEM associated with a repeat episode, neuromyelitis optica, CIS, and pediatric multiple sclerosis (MS).[2] It is important to distinguish transient demyelinating syndromes from the life-long diseases like MS. ADEM and CIS are the commonest forms of CIDD. Amongst the CIDD, multiple sclerosis and neuromyelitis optica are rare in children especially in India as is also evident from the present study.[3]

Acute disseminated encephalomyelitis is a monophasic inflammatory demyelinating disorder of the CNS that occurs after vaccination or systemic viral infections or that may even be idiopathic. According to the consensus definition, to diagnose ADEM the clinical presentation must be polysymptomatic and must include encephalopathy (either behavioral change in the form of confusion, irritability, or alteration in consciousness). CIS also results from a presumed inflammatory demyelinating cause and the clinical event may either be monofocal or multifocal but usually does not include encephalopathy (except in cases of brainstem syndromes). Examples of CIS include conditions like optic neuritis (unilateral or bilateral); transverse myelitis; brainstem, cerebellar, and/or hemispheric dysfunction.[1]

Clinically evident antecedent infection occurs in 2/3rds of children[3] as seen in the present study. ADEM affects predominantly children and young adults. Relapses though rare are known making its distinction from multiple sclerosis difficult. New event of ADEM with a recurrence of the initial symptoms and signs, 3 or more months after the first ADEM event, without involvement of new clinical areas by history, examination, or neuroimaging is called as recurrent ADEM and new clinical event when it involves new anatomic areas of the CNS is called as multiphasic ADEM.[1] Neurologic symptoms usually follow between 1 and 20 days of infectious illness or vaccination. Clinical spectrum of ADEM/CIS is wide with mild subclinical disease at one end and a fulminant presentation with seizure, coma and death secondary to hemorrhagic acute disseminated encephalomyelitis at the other end. It can involve cerebral hemisphere (hemiparesis, aphasia), brain stem (cranial nerve palsies, ataxia) and spinal cord (paraparesis with or without bowel and bladder involvement).[4] Symptoms and signs usually include motor weakness, paraesthesias, spasticity, dysarthria, obtundation, headaches, and ataxia but the disease course can progress to seizures, myoclonus, meningism especially in acute haemorrhagic form of ADEM.[5]

Many conditions have been reported to precede ADEM. These include viral infections like mumps, coxsackie, influenza, Ebstein Barr virus, human immunodeficiency virus, herpes simplex virus, human herpes virus 6, hepatitis A, B, measles, rubella, varicella, cytomegalovirus, Japanese B; bacterial infections like group A ß hemolytic streptococci, campylobacter jejuni, salmonella, Chlamydia, mycoplasma, legionella, leptospira, Borrelia, and rickettsiae; fungal infections. It is also associated with vaccination against influenza, measles, mumps, pertussis, tetanus, meningoccal disease, Japanese B, BCG and rabies; organ transplant and drugs like gold, serum administration.[4,5] Illness is referred to as post infectious, parainfectious, post exanthematous, and post vaccineal in reference to the clinical setting in which it can occur.[4] ADEM/CIS are thought to be auto immune diseases. Myelin auto antigens such as myelin basic protein, proteolipid protein, and myelin oligodendrocyte protein could share similar antigenic determinants with those on an infecting pathogen.[2]

The results of the present study correlate well with the findings of case series published by Hynson *et al*., Tenembaum *et al*. and others.[6,7] Most of the cases with ADEM were referred with a diagnosis of encephalitis and few of them were even on antiviral drug as well. This highlights the importance of correct diagnosis. Under recognition of this entity could be because of lack of awareness among pediatricians and paucity of investigative facilities like MRI in most centers catering to children in developing countries. Appropriate diagnosis after imaging helps in prognostication and planning the management.

A cranial CT scan of the brain may be normal, so is often not helpful in establishing a diagnosis. However, MRI of the brain may show early subtle features of the disseminated CNS demyelination associated with ADEM.[5] MRI T2-weighted, and FLAIR images show the abnormalities more readily than T1-weighted images. These changes are usually distinguishable from MS.[8] Involvement of the deep and subcortical white matter is almost universal, whereas grey matter lesions are seen less often, and only in addition to the more characteristic white matter lesions. Involvement of the thalami and basal ganglia is a typical finding in ADEM, but unusual in MS and may be a useful marker in its differentiation. In ADEM supratentorial lesions tend to be asymmetrical, whereas thalamic and basal ganglia lesions are often symmetrical. In addition, the lesions may be extensively distributed. Follow-up MRI scans will show evidence of partial or complete resolution of the lesions and may be useful in differentiating monophasic from multiphasic disease. Thus, MRI plays an important role and is more sensitive than CT scan in picking up the lesions. Other techniques not widely available, but helpful in supporting the diagnosis of ADEM, are gadolinium diethylenetriamine penta-acetic acid (Gd-DTPA) enhancement of the lesions on MRI, and areas of increased activity on single photon emission computed tomography.[9,10]

Treatment of CIDD is not clear with different groups using different modes of therapy.[1,2] Spontaneous remission can occur, although it seems likely that the current practice of active treatment with steroids is usually beneficial.[11] Though many have used pulse steroids, there is role for use of intravenous immunoglobulin and plasmapheresis in severe cases of CIDD not responding to steroids or in those where use of steroids can be harmful.[12–15] In order to know the correct presentation and management of CIDD, there is a need to use standard definitions like that proposed by the consensus group so as to have evidence based guidelines for individual disorders.[15] In the present study also patients responded well to both methylprednisolone and dexamethasone. Because of small numbers head to head comparison could not be done. But the present study suggests that patients who cannot afford methylprednisolone can be given a trial of high dose dexamethasone.

Most children with ADEM present with an acute aggressive encephalopathy with multifocal neurological deficits. Most make excellent progress over the following days, weeks, or months with no subsequent neurological impairment. A minority of children are left with a neurological impairment that can range from mild to severe. This can include motor disability, visual impairment, cognitive impairment, behavioral impairment, and epilepsy. Any neurological impairment will need to be addressed, and appropriate coordinated multiagency rehabilitation organised.[5,16]

## Conclusions

CNS inflammatory demyelinating disorders are not uncommon in developing countries. It affects both sexes equally and is a disease with good prognosis in more than 80% patients. Mortality is rare and depends on the extent of lesions, timely diagnosis, and management. Neuroimaging in the form of MRI is essential for diagnosis. Multiple sclerosis and neuromyelitis optica are uncommon in Indian children. Large studies from different centers are required to further justify these facts.
